# Metachronous colorectal liver metastasis that occurred 10 years after laparoscopic colectomy: a case report

**DOI:** 10.1186/s40792-022-01503-9

**Published:** 2022-08-01

**Authors:** Hidetoshi Shidahara, Tomoyuki Abe, Akihiko Oshita, Yusuke Sumi, Hiroshi Okuda, Manabu Kurayoshi, Shuji Yonehara, Tsuyoshi Kobayashi, Hideki Ohdan, Toshio Noriyuki, Masahiro Nakahara

**Affiliations:** 1 Department of Surgery, Onomichi General Hospital, Onomichi, Hiroshima Japan; 2 Department of Pathology, Onomichi General Hospital, Onomichi, Hiroshima Japan; 3 Department of Gastroenterological and Transplant Surgery, Graduate School of Biomedical and Health Sciences, Hiroshima University, Hiroshima, Japan

**Keywords:** Colorectal liver metastasis, Metachronous, Delayed onset

## Abstract

**Background:**

Delayed onset of colorectal liver metastasis (CRLM) > 5 years after primary colorectal surgery is rare. Herein, we report a case of delayed-onset CRLM that occurred 10 years after primary surgery, for which laparoscopic hepatectomy was performed.

**Case presentation:**

A 68-year-old man was admitted to the hospital. His medical history revealed double colon cancer detected 10 years ago, for which laparoscopic colectomy was performed. The pathological tumor–node–metastasis stages were stages I and II. Thereafter, oral floor cancer occurred 7 years after the primary surgery and was curatively resected. The annual follow-up with positron emission tomography–computed tomography (CT) identified a tumor at segment 7/8 (S7/8) of the liver with an abnormal accumulation of fluorodeoxyglucose. Dynamic CT showed a 23-mm tumor, with ring enhancement in the early phase. Magnetic resonance imaging with gadolinium–ethoxybenzyl-diethylenetriamine penta-acetic acid demonstrated that the tumor had high intensity in T2 weighted sequences and low intensity in the hepatobiliary phase. With a preoperative diagnosis of intrahepatic cholangiocarcinoma or delayed liver metastasis, laparoscopic S7/8 partial resection was performed. The operative time was 324 min, and the intraoperative bleeding volume was 35 mL. The patient was discharged on day 15 without any postoperative complications. Upon histopathological examination, the final diagnosis was CRLM. The patient has survived for 1 year without any recurrence.

**Conclusions:**

It is important to pay attention to the occurrence of delayed-metachronous CRLM.

## Background

Colorectal cancer (CRC) is the most common cancer in Japan, accounting for 16% of all new cancer diagnoses [1]. Prognosis after curative surgery for CRC has a 5-year survival rate of approximately 80% [2]. However, colorectal liver metastasis (CRLM) frequently occurs after primary resection, and the gold-standard treatment for CRLM is hepatectomy under certain conditions [3–5]. Perioperative systematic chemotherapy for resectable CRLM is not yet an established treatment [6]. The prognostic factors for the recurrence-free survival in patients with CRLM are preoperative carcinoembryonic antigen (CEA) level, carbohydrate antigen 19-9 (CA19-9) level, tumor diameter, tumor number, poorly differentiated primary CRC, primary lymph node metastasis, extrahepatic metastasis, and synchronous metastatic pattern [7, 8].

Most cases of CRLM are synchronously detected; however, metachronous CRLM has been reported to have an incidence of 9.4–12.9% [9, 10]. Delayed CRLM occurring 5 years after primary tumor resection has rarely been reported until now. We encountered a case of metachronous CRLM that occurred 10 years after primary tumor resection. Here, we report a case with a literature review.

## Case presentation

**Fig. 1:**
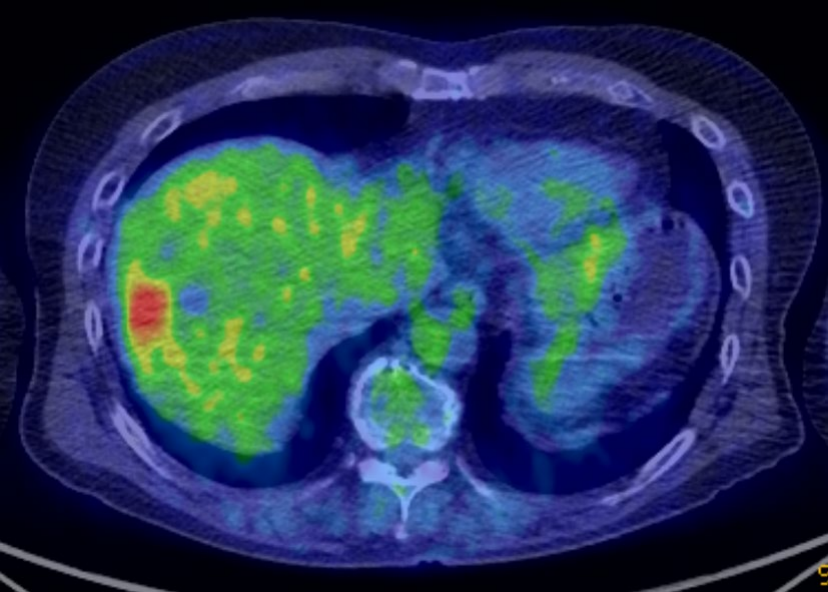
Positron emission tomography–computed tomography shows a tumor with abnormal accumulation of fluorodeoxyglucose at liver S7/8

**Fig. 2:**
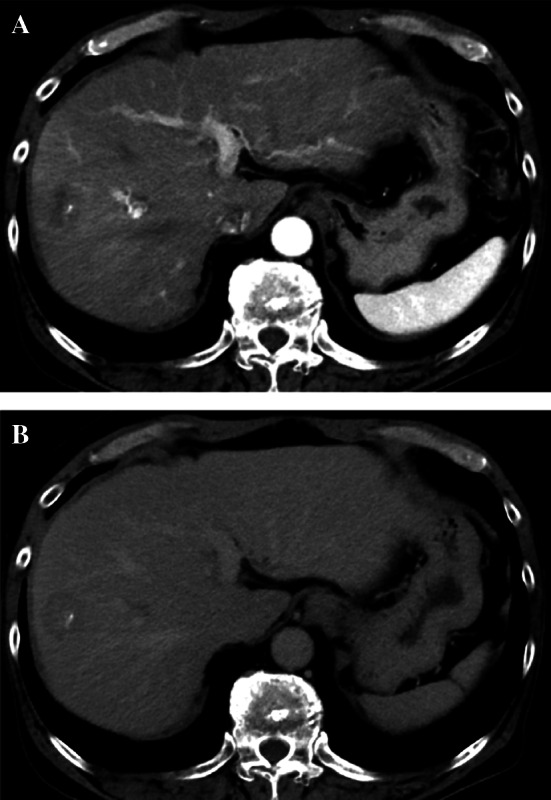
**A**, **B** Abdominal contrast-enhanced computed tomography shows a tumor measuring 23 mm in size with ring enhancement in the early phase (arrow). The tumor shows hypointensity in the delayed phase (arrow)

**Fig. 3:**
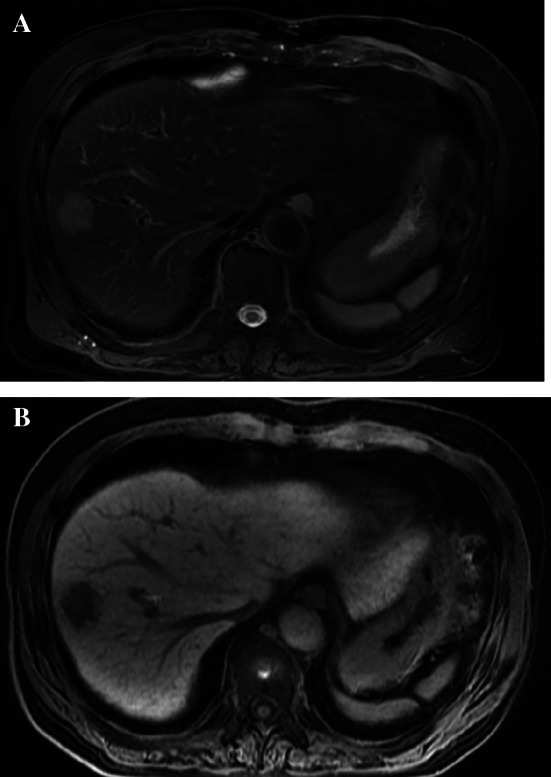
**A**, **B** Gadolinium–ethoxybenzyl-diethylenetriamine penta-acetic acid-enhanced magnetic resonance imaging showing a tumor as a hyperintense lesion without central filling on the T2-weighted image (arrow) and a hypointense lesion in the hepatobiliary phase (arrow)

A 68-year-old man with a history of transverse colon cancer (tub2, ly1, v0, pT1N0M0 stage I) and sigmoid colon cancer (pap, ly0, v0, pT3N0M0 stage IIA) was admitted to our hospital. Curative laparoscopic surgery was performed for synchronous double colon cancer 10 years ago. Conventional surveillance was performed 5 years after the primary colon cancer. The CEA level in the fifth year was 12.6 ng/mL. His CEA level remained slightly elevated due to heavy smoking. Oral floor cancer was detected seven years after surgery. Contrast-enhanced computed tomography (CT) revealed no space-occupying lesions in the liver. The squamous cell carcinoma (SCC) antigen was examined; however, CEA levels were not measured. Marginal mandibulectomy was performed at the Department of Otolaryngology of our hospital. The final diagnosis was SCC (pT1N0M0 stage I). After another 3 years, a liver tumor was detected during annual surveillance for oral floor cancer. Positron emission tomography–CT showed that the tumor was located on segment 7/8 (S7/8) of the liver, with an abnormal accumulation of fluorodeoxyglucose (Fig. 1). Dynamic CT showed that the tumor was 23 mm in size, with ring enhancement in the early phase (Fig. 2A). In the delayed phase, the tumor had a low-density area (Fig. 2B). Gadolinium–ethoxybenzyl-diethylenetriamine penta-acetic acid-enhanced magnetic resonance imaging demonstrated a hyperintense lesion without central filling in T2-weighted image sequences and a hypointense lesion in the hepatobiliary phase (Fig. 3A, B). In addition to a solitary liver tumor, no extrahepatic metastases were detected. Laboratory data showed a highly elevated CEA level (29.9 ng/mL). The levels of other tumor markers, including CA19-9, α-fetoprotein, and protein induced by vitamin K absence or antagonist II, were within normal limits. Tumor biopsy provided a diagnosis of moderately differentiated tubular adenocarcinoma. The preoperative differential diagnoses included delayed-onset CRLM, intrahepatic cholangiocarcinoma, and liver metastasis from oral floor cancer.

**Fig. 4:**
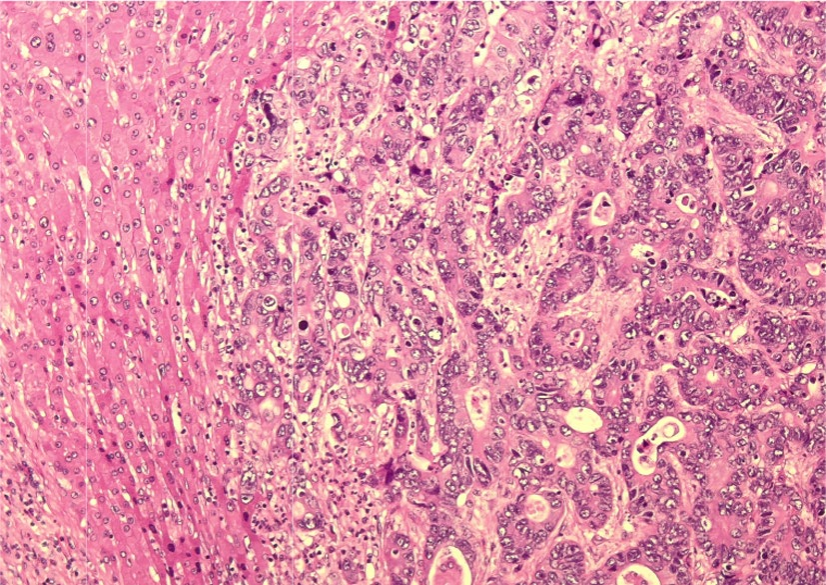
Histological examination reveals tall columnar tumor cells forming small glandular duct structures. The pathological diagnosis is moderately differentiated tubular adenocarcinoma (hematoxylin and eosin staining, ×10)

**Fig. 5:**
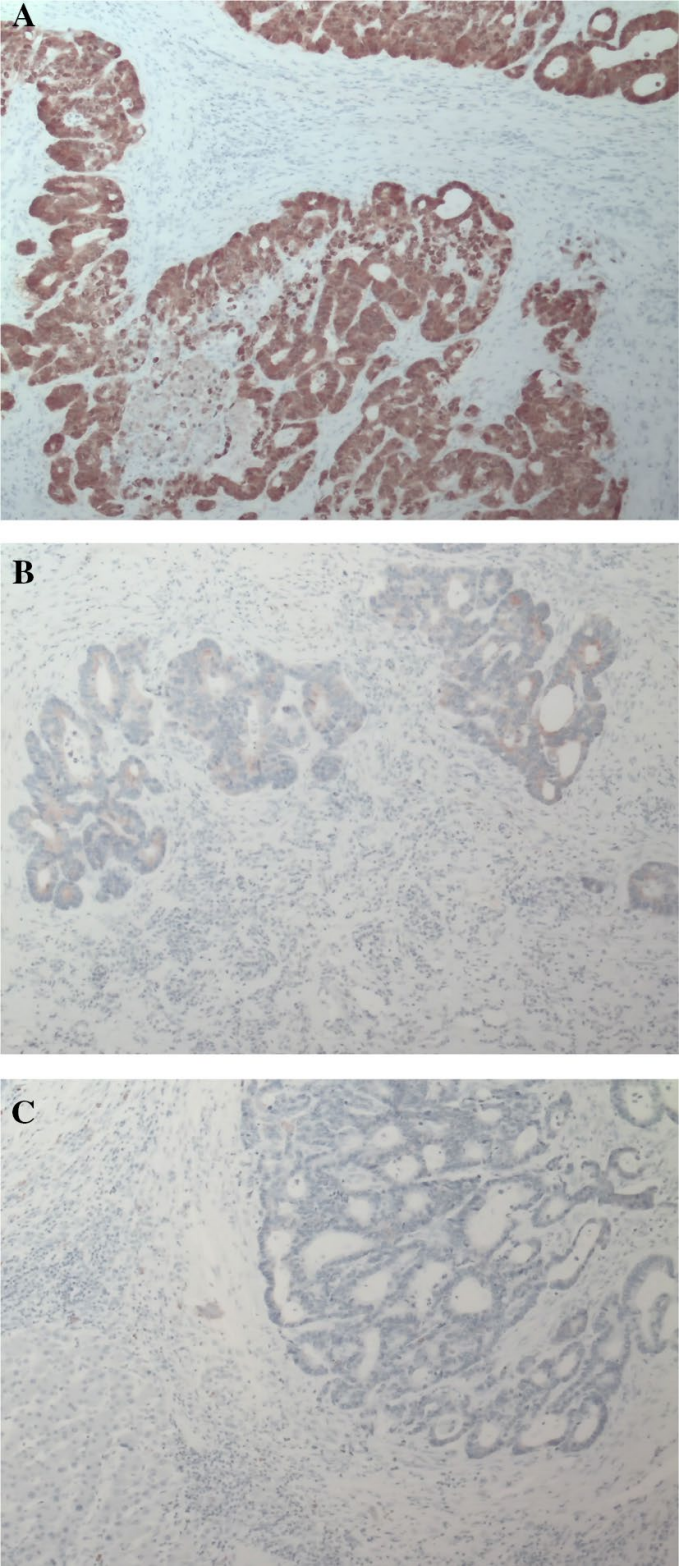
**A**–**C** Immunohistochemical analysis revealed that the tumor was positive for caudal-type homeobox protein 2 and negative for CK7 and CK20. (Staining, ×10)

A laparoscopic S7/8 partial resection was performed. The operative time was 324 min, and the intraoperative bleeding volume was 35 mL. The histopathological diagnosis was moderately differentiated tubular adenocarcinoma (Fig. 4). Immunohistochemical analysis revealed that the tumor was positive for caudal-type homeobox protein 2 (Fig. 5A) and negative for cytokeratin (CK) 7 (Fig. 5B) and CK20 (Fig. 5C). Therefore, a pathological diagnosis of CRLM was made. The patient has survived for 1 year without any recurrence.

## Discussion

Most CRC recurrences occur synchronously or within 5 years of primary surgery. The distant metastatic organs were the liver, lungs, and peritoneum. The incidence of delayed distant recurrence 5 years after primary surgery for CRC was < 1% (0.63% in a Japanese study [2]). To date, five cases of delayed metachronous CRLM occurring > 5 years after primary surgery have been reported (Table 1) [11–15]. All patients were men, and the primary tumor was well to moderately differentiated tubular adenocarcinoma (tub1–2) on histopathological examination. CRLM mostly occurs in the right lobe of the liver as a solitary tumor. In particular, the tumor doubling time in our case is estimated to be approximately 81 days, which was calculated based on the tumor growth. The tumor doubling time was calculated using the following formula: (day 2 value − day 1 value)/10*(logD2 − logD1), *D: diameter. Compared to 6 months prior to hepatectomy, tumor diameter had approximately doubled in size from 14 to 23 mm at the time of resection. Slow growth patterns could contribute to the good prognosis of this disease. The prognosis after hepatectomy was satisfactory, and five patients, including ours, survived without any recurrence. Notably, all cases had a relatively early tumor–node–metastasis stages (stage I or II). One report demonstrated that not only a primary tumor stage of less than stage II but also microlymphovascular invasion could be important factors for delayed local and distant metastases from CRC [15].

**Table 1:** Characteristics of patients with delayed metachronous CRLM treated with hepatectomy

Case	Age (years)	Sex	Lesion	pTN	TNM stage	Primary tumor differentiation	Lymphovascular invasion	Interval from primary tumor to CRLM (months)	Tumor number	Tumor location	Recurrence-free survival from hepatectomy (months)	Status	Cause of death
1	80	M	Rs	pT3 N1	IIIa	tub1	ly0, v1	60	1	S5	9	Dead	Other (SAH)
2	76	M	S	pT1b N0	I	tub1	ly2, v1	98	2	S5, S7	u/a	u/a	–
3	56	M	A	pT3 N0	II	tub2	ly0, v0	112	1	S4/5	27	Alive	–
4	58	M	S	pT3 N0	II	tub2	ly0, v0	84	1	S6/7	24	Alive	–
5	77	M	Rs	pT3 N0	II	tub2	ly2, v2	132	1	S7	18	Alive	–
Our case	68	M	T, S	pT1N0 and pT3N0	I,II	tub2 and pap	ly1, v0 and ly0, v0	120	1	S7/8	12	Alive	–

A: ascending colon; M: male; N: nodes; Rs: rectosigmoid colon; SAH: subarachnoid hemorrhage; S: sigmoid colon; T: transverse colon; TNM: tumor–node–metastasis

Whether patients with CRLM should receive neoadjuvant chemotherapy (NAC), particularly in cases of resectable CRLM, remains controversial. Some studies have reported that upfront surgery is recommended for resectable CRLM, because the prognosis of upfront surgery is equivalent to that of NAC [16, 17]. Others demonstrated that NAC could provide prognostic benefits in selected patients with bulky tumors, multiple CRLMs, and elevated CEA levels [18, 19]. Conversely, adjuvant chemotherapy (ACT) after resection of CRLM is generally recommended, because ACT improves 3-year recurrence-free survival [20, 21]. In particular, ACT could provide survival benefits in selected patients with synchronous CRLM and early onset metachronous CRLM [22]. Further studies are required to establish the benefits of systemic chemotherapy in CRLM patients.

Oligometastatic surgery is effective for various types of cancer. Among them, pancreatic metastasis from renal cell carcinoma is indicated for surgical resection because of the good long-term prognosis after metastasectomy [23, 24]. Metachronous pulmonary metastasis from pancreatic ductal adenocarcinoma and delayed-onset liver metastasis from gastrointestinal submucosal tumors are also good candidates for oligometastatic surgery [25, 26]. In the setting of pulmonary oligometastasis from colorectal cancer, surgical resection would provide a good prognosis comparable to that of liver metastasis. A long disease-free interval (≥ 36 months from primary CRC), solitary nodule, and normal pre-thoracotomy CEA level (< 5 ng/mL) are known to be independent prognostic factors [27]. Theoretically, the malignant potential of distant metastatic tumors can be dramatically lower than that of primary tumors owing to the intercellular interaction of tumor cells (seed) and the microenvironment (soil) of the metastasized organ [28, 29], a phenomenon that is supported by the well-known “seed and soil hypothesis.” Therefore, oligometastatic surgery would be beneficial, even if the primary tumor has high malignant potential.

## Conclusions

We report a case of delayed-metachronous CRLM. It is important to pay attention to the occurrence of delayed-metachronous CRLM.

## Data Availability

Data sharing is not applicable to this article as no data sets were generated or analyzed during the current study.

## Abbreviations

ACT: Adjuvant chemotherapy
CA19-9: Carbohydrate antigen 19-9
CEA: Carcinoembryonic antigen
CK: Cytokeratin
CRC: Colorectal cancer
CRLM: Colorectal liver metastasis
CT: Computed tomography
NAC: Neoadjuvant chemotherapy
SCC: Squamous cell carcinoma
S7/8: Segment 7/8
